# Novel strong tissue specific promoter for gene expression in human germ cells

**DOI:** 10.1186/1472-6750-10-58

**Published:** 2010-08-17

**Authors:** Denis Kuzmin, Elena Gogvadze, Roman Kholodenko, Dawid P Grzela, Maxim Mityaev, Tatyana Vinogradova, Eugene Kopantzev, Galina Malakhova, Maria Suntsova, Dmitry Sokov, Zoltán Ivics, Anton Buzdin

**Affiliations:** 1 Shemyakin-Ovchinnikov Institute of Bioorganic Chemistry, Moscow, Russia; 2 Max Delbrück Center for Molecular Medicine, Berlin, Germany; 3 P.A. Herzen Moscow Oncological Research Institute, Moscow, Russia

## Abstract

**Background:**

Tissue specific promoters may be utilized for a variety of applications, including programmed gene expression in cell types, tissues and organs of interest, for developing different cell culture models or for use in gene therapy. We report a novel, tissue-specific promoter that was identified and engineered from the native upstream regulatory region of the human gene *NDUFV1 *containing an endogenous retroviral sequence.

**Results:**

Among seven established human cell lines and five primary cultures, this modified *NDUFV1 *upstream sequence (mNUS) was active only in human undifferentiated germ-derived cells (lines Tera-1 and EP2102), where it demonstrated high promoter activity (~twice greater than that of the SV40 early promoter, and comparable to the routinely used cytomegaloviral promoter). To investigate the potential applicability of the mNUS promoter for biotechnological needs, a construct carrying a recombinant cytosine deaminase (RCD) suicide gene under the control of mNUS was tested in cell lines of different tissue origin. High cytotoxic effect of RCD with a cell-death rate ~60% was observed only in germ-derived cells (Tera-1), whereas no effect was seen in a somatic, kidney-derived control cell line (HEK293). In further experiments, we tested mNUS-driven expression of a hyperactive *Sleeping Beauty *transposase (SB100X). The mNUS-SB100X construct mediated stable transgene insertions exclusively in germ-derived cells, thereby providing further evidence of tissue-specificity of the mNUS promoter.

**Conclusions:**

We conclude that mNUS may be used as an efficient promoter for tissue-specific gene expression in human germ-derived cells in many applications. Our data also suggest that the 91 bp-long sequence located exactly upstream *NDUFV1 *transcriptional start site plays a crucial role in the activity of this gene promoter *in vitro *in the majority of tested cell types (10/12), and an important role - in the rest two cell lines.

## Background

Tissue-specific promoters may be utilized for a variety of applications, including programmed gene expression in cell types, tissues and organs of interest, and for developing different cell culture models or for use in gene therapy. For example, one of the most promising approaches of gene therapy is the delivery of "suicide" genes under transcriptional control of promoters highly active in cancer cells (e.g, [1,2]). In therapeutic constructs it is extremely important to precisely tune the transcriptional activity of the gene expression system in order to ensure the safety of a gene-therapeutic drug for normal tissues. To reach this goal, native promoter sequences are frequently modified by deleting or adding different regulatory motifs, most frequently - transcription factor recognition sites [3].

Among the suicide genes, the most efficient are those that have a "bystander" effect, *i.e*. activity not only for the cells that received the gene construct, but also for the neighboring cells. The bystander effect is especially valuable when the efficiency of gene delivery into the cell nuclei is low, as it is the case for a number of human tissues [4]. This makes it possible that even a small number of transfected cells expressing a therapeutic construct may cause massive target cell death in a malignant tissue [5]. However, fine tuning of gene activity is more precise in binary systems that may include a suicide gene product (an enzyme) and its chemical substrate that together elicit a cytotoxic effect. In this system, both the gene product and the substrate are harmless when present separately in the cell. However, codelivery of the enzyme and its substrate results in conversion of the substrate into a toxic metabolite that kills the cell. Several, efficient binary systems have been developed to date, for example, herpes simplex virus thymidine kinase with gancyclovir [6], or cytosine deaminase with 5-fluoro cytosine [7].

In this paper we describe a novel, genetically engineered, tissue-specific promoter and propose two, related gene cassettes for generation of gene therapeutic constructs.

Previous studies suggested that the long terminal repeats (LTRs) of human endogenous retroviruses exhibit significant enhancer activity *in vitro *[8,9]. For the HERV-K (HML-2) elements, this effect was especially strong in cultured human testicular germ cells, in good agreement with numerous reports documenting high transcriptional activities of the HERV-K (HML-2) elements in germ cell-derived cancer tissues (e.g., [10-15]). Many HERV-K (HML-2) genomic inserts are located in upstream regions of genes close to transcriptional start sites, and theoretically may serve as functional enhancer elements *in vivo *[12,16]. We hypothesized that removing non - HERV-K (HML-2)-associated regulatory elements from the upstream regions of these genes might switch their promoter specificities towards relatively higher expression in germ cells. We have tested this hypothesis on the human gene *NDUFV1 *that has a HERV-K (HML-2) LTR relatively close to its transcriptional start site (distance ~2.6 kb). Considering that important regulatory sequences are frequently conserved, we removed all conserved motifs from the *NDUFV1 *upstream region.

Here we show that the resulting modified *NDUFV1 *upstream sequence (mNUS) may serve as a strong, tissue-specific promoter. On a panel of twelve human cell lines we show that mNUS provides strong, selective gene expression in testicular, germ-derived cells. This specificity was further supported by a selective cytotoxic effect of an mNUS-driven suicide gene (RCD, recombinant cytosine deaminase), only in cultured germ-derived cells. Finally, transposase expression under transcriptional control of mNUS, provided selective, germ cell-specific genomic *Sleeping Beauty *transposition. Our data suggest that mNUS may be used as an efficient, tissue-specific promoter for germ cells.

## Results

### Modification of *NDUFV1 *upstream sequence

*NDUFV1 *encodes the mitochondrial protein NADH dehydrogenase ubiquinone flavoprotein 1 and is expressed in all human tissues as a housekeeping gene [17]. At the distance of ~2.6 kb upstream of the *NDUFV1 *transcriptional start site there is a HERV-K (HML-2) endogenous retroviral solitary LTR element. Bioinformatical screening has revealed a highly conserved, 91 bp-long sequence exactly upstream the *NDUFV1 *transcriptional start site (Additional file 1, Fig. S1). In order to test the possibility that removal of this conserved sequence may alter tissue specificity of the *NDUFV1 *upstream region, we have PCR-amplified and cloned two genomic fragments, one including the whole, 3665 bp-long upstream sequence, and the other, 3574 bp-long sequence lacking the conserved region. Primary structure of both cloned fragments was confirmed by sequencing. The shorter sequence termed *modified NDUFV1 upstream sequence *(mNUS) was further compared with the native upstream region (NUS; the longer sequence) with respect to transcriptional activity in functional tests.

### Tissue specificity of mNUS promoter

Promoter activity of mNUS was tested using the *pGL3 basic *vector (Promega) that includes the firefly luciferase reporter gene, but lacks any promoter and enhancer elements. We cloned mNUS and NUS into *pGL3 basic *upstream of the reporter gene. For functional tests we chose seven established human cell lines and four primary cell cultures of different origin. Cell lines were: Tera-1, EP2102, Tera-2 (testicular germ cells), NGP127 (neuroblastoma), HepG2 (hepatocarcinoma), A549 (lung carcinoma) and HEK293 (transformed embryonic kidney cells). Primary human cell cultures included macrophages, umbilical cord mesenchymal stem cells (UC-MSC), placental mesenchymal stem cells (PMSC) and bone marrow mesenchymal stem cells (BM).

**Figure 1:**
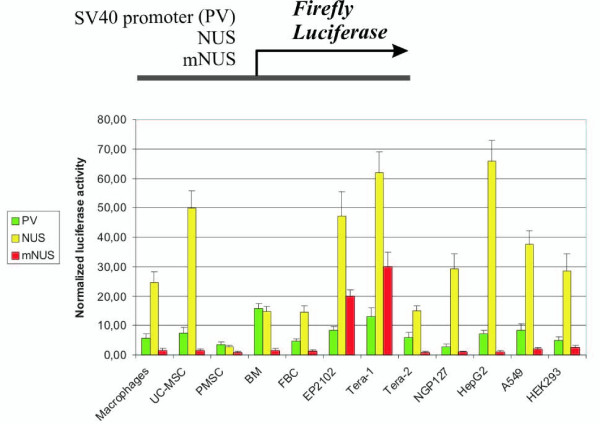
**Luciferase reporter assay of the promoter activities of SV40, NUS and mNUS elements in twelve human cell cultures**. The column height reflects the mean value of relative luciferase activity from at least four transfections, and error bars indicate standard error. Cell lines were: Tera-1, EP2102, Tera-2 (testicular germ cells), NGP127 (neuroblastoma), HepG2 (hepatocarcinoma), A549 (lung carcinoma) and HEK293 (transformed embryonic kidney cells). Primary human cell cultures included macrophages, umbilical cord mesenchymal stem cells (UC-MSC), placental mesenchymal stem cells (PMSC) and bone marrow mesenchymal stem cells (BM).

NUS and mNUS promoter activities were measured using "Dual luciferase" system in a series of transient transfection experiments (Fig. 1). To minimize errors caused by the differences in transfection efficiencies in the independent replicates, reporter *firefly *luciferase activity was normalized to the activity of herpes simplex virus promoter-driven *renilla *luciferase expressed from the cotransfected plasmid pRL-TK that provides relatively uniform levels of *renilla *luciferase expression in cell types of different origin (Additional file 2, Fig. S2). We compared NUS/mNUS and virus SV40 promoter activities.

The intact sequence (NUS) displayed high promoter activity in the majority of tested cell types (Fig. 1). In most of cell cultures (10 out of 12 lines), NUS activity was significantly higher than that of the SV40 promoter (Fig. 1). However, a very different pattern was observed for the modified upstream sequence (mNUS)that showed a sharp decrease in all cell types, except in Tera-1 and EP2102 (Fig. 1). For example, relative to NUS, mNUS activity has diminished ~65-fold in HepG2, ~35-, 33-, 19- fold in UC-MSC, NGP127, Tera-2, but only about 2-fold in Tera-1 and EP2102 cells. In the absolute values, in Tera-1 cells mNUS activity was about twice as high as that of the SV40 promoter, whereas for the other cell types it was hardly detectable. These data suggest that the 91 bp-long sequence located just upstream of the *NDUFV1 *transcriptional start site plays a key role in the activity of this gene promoter *in vitro *in the majority of tested cell types. Interestingly, there was a remarkable difference between mNUS promoter activities in Tera-1, EP2102 and Tera-2 cells: in contrast to high values in Tera-1 and EP2102, it was far lower in Tera-2. Although all three cell lines have been established from testicular germ cell tumors, Tera-1 and EP2102 cells represent undifferentiated germ cell precursors, whereas Tera-2 cells are derived from partially differentiated cells [18].

These results suggest that mNUS is a highly tissue-specific promoter. Its activity is relatively high in undifferentiated testicular germ cells, and negligible in all other cell types tested. In further experiments, we aimed to investigate the potential value of mNUS for biotechnological applications.

### Functional tests of mNUS-RCD in suicide gene therapy assays

In order to estimate the potential value of mNUS for suicide gene therapy, we designed a construct having a suicide gene under transcriptional control of mNUS promoter. As a suicide gene, the recombinant bifunctional yeast cytosine deaminase (CD) previously engineered by Erbs and co-authors [19] was chosen. CD normally converts cytosine into uracil. However, this enzyme also deaminates the nontoxic prodrug 5-fluorocytosine (5FC) to the highly toxic chemotherapeutic drug 5-fruorouracil. The latter molecule is converted by the cellular enzymatic machinery to 5-fluoro-UTP and 5-fluoro-dUMP. 5-fluoro-UTP is included into RNA molecules, which results in the inhibition of the nuclear processing of the ribosomal and mRNAs, whereas 5-fluoro-dUMP irreversibly inhibits thymidylate synthase, preventing DNA synthesis. Finally, CD cytotoxicity is greatly amplified due to bystander effect [20].

Recombinant CD (RCD) was cloned into plasmid vectors containing the tissue-specific mNUS or constitutive cytomegaloviral (CMV) promoters or a vector lacking any promoter and enhancer elements that was used as negative control in functional tests. RCD gene activity was pre-tested using no-RCD and CMV-RCD constructs in Tera-1 cells, and RCD expression was visualized by Western blot analysis. CMV promoter was chosen for positive control experiments because it was highly active in our tests in both Tera-1 and HEK 293 cell lines (Additional file 3, Fig. S3).

**Figure 2:**
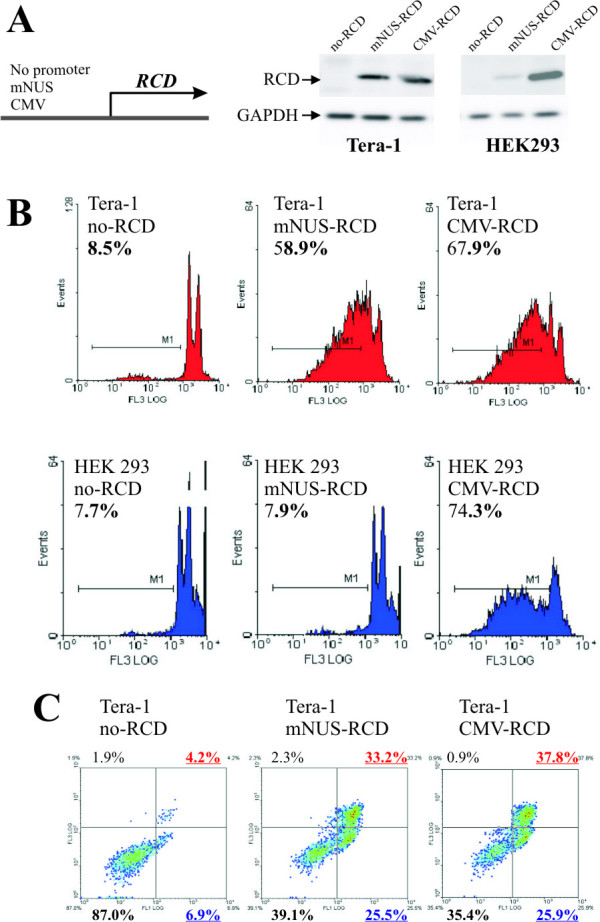
**Effect of mNUS-RCD cassette on cell viability**. Cells were transfected with no-RCD (negative control), mNUS-RCD (analyte) and CMV-RCD (positive control) constructs and grown on 5FC-containing medium for 24 hours. Transfection efficiencies were measured using cotransfection with GFP-encoding vector TurboGFP (Evrogen), only transfections with efficiency values 65-80% were scored. (A), representative Western blot results with the antibodies specific to RCD and to a housekeeping protein GAPDH. (B), representative results of the propidium iodide (PI) assay. Histograms for Tera-1 cells are shown in red, for HEK 293 - in blue. Proportions of dead cells are given in percents. (C), representative FACS data for the Annexin V assay. Incidences of the living Tera-1 cells are shown in lower left quadrant (proportion in percents - bottom, left), of apoptotic cells - in lower right quadrant (proportion - bottom, right), of necrotic cells - in upper right quadrant (proportion - top, right). Overall proportions of dead cells were calculated as the total of proportions of apoptotic and necrotic cells.

Along with untransfected controls, part of the transfected cells was exposed to 5-fluorocytosine (5FC), another part did not receive 5FC. Forty-eight hours after addition of 5FC, cell viability was measured using propidium iodide (PI) tests. As expected, there was an overall correlation between the RCD expression and observed cytotoxicity (Fig. 2A). The proportion of dead cells was almost equal for untransfected Tera-1 cells both exposed and not exposed to 5FC, and for the no-RCD Tera-1 transfectants (~9-10%). However, in the exposed CMV-RCD transfectants ~65% of the cells died, as compared to ~10% in unexposed CMV-RCD cells. Therefore, in good agreement with published data [19,20], cytotoxic activity of RCD completely depended on the presence of 5FC in our tests. Furthermore, RCD activity was promoter-dependent as in the promoter-less construct there was no toxic effect, whereas the CMV-RCD construct was highly toxic for the +5FC (5FC added) cells. Importantly, in our system there was no promoter "leakage" to produce background cytotoxicity. Finally, 5FC itself was not toxic, whereas in combination with RCD its product was efficiently killing the cells.

Functional tests of mNUS-RCD were performed on Tera-1 and HEK293 cells. In the previous assay, the highest activity of mNUS-driven luciferase was observed in germ cancer-derived cells (lines Tera-1 and EP2102; Fig. 1), whereas reporter gene expression was significantly lower in other cell cultures. For testing tissue-specificity of mNUS-RCD, HEK293 cells were used as a control as they were demonstrating the highest mNUS-driven expression except for Tera-1 and EP2102 cells (Fig. 1). Cells were transiently transfected with the mNUS-RCD, positive control CMV-RCD and negative control no-RCD expression plasmids and then grown in +5FC medium (Fig. 2; shown for 24-hour application). 24 and 48 hours after addition of the 5FC, cell viability was measured using the Propidium iodide (PI) and Annexin V tests. In our experiments, both assays gave similar results (Table 1).

**Table 1:** Cell viability tests on Tera-1 and HEK-293 cells.

**Experiment**^**a**^	Tera-1		HEK-293	
	**PI assay**^**b**^	**Annexin V**^**c**^	**PI assay**^**b**^	**Annexin V**^**c**^

-5FC; -Transf.	9.2 ± 2.3	ND	7.7 ± 1.5	ND

-5FC; no-RCD	9.0 ± 1.7	ND	7.4 ± 1.7	ND

-5FC; mNUS-RCD	8.8 ± 2.9	ND	7.8 ± 2.1	ND

-5FC; CMV-RCD	9.9 ± 2.5	ND	8.2 ± 2.2	ND

+5FC; -Transf.	9.4 ± 2.1	10.9 ± 3.3	7.4 ± 1.8	8.7 ± 2.3

+5FC; no-RCD	9.1 ± 2.5	10.7 ± 2.6	7.7 ± 1.6	8.6 ± 2.9

+5FC; mNUS-RCD	59.5 ± 6.4	59.1 ± 3.9	7.8 ± 1.7	8.4 ± 2.8

+5FC; CMV-RCD	67.3 ± 7.5	64.9 ± 5.6	73.8 ± 11.3	75.5 ± 8.6

^a^Cell viability tests shown on this table were done 24 hours after application of 5FC. "-5FC" means "no 5FC was added", "+FC" marks experiments with the addition of 5FC to the medium. "-Transf.", "no-RCD", "mNUS-RCD" and "CMV-RCD" mean "no transfection", "transfection with *no-RCD *construct", "transfection with *mNUS-RCD*" and "transfection with *CMV-RCD*", accordingly.

^b^Propidium iodide tests were done at least in quadruplicates. The data represent integral proportion of necrotic and apoptotic cells.

^c^Annexin V assay was performed for all the experiments with the addition of 5FC to the medium. Tests were done in triplicates. The data represent integral proportion of necrotic and apoptotic cells.

For Tera-1, we observed background numbers (~9%) of dead cells for all the transfectants grown in -5FC medium, very similar values to negative controls grown in the +5FC medium. In the positive controls (+5FC), cell death rate was ~65-67%. Finally, in mNUS-RCD transfectants (+5FC), we observed ~59-60% of dead cells.

In HEK293, all transfectants grown in -FC conditions and negative controls also displayed background proportion of dead cells (~7-9%), in contrast to high values in positive controls (~74-76%). In mNUS-RCD transfectants (+5FC), the number of dead HEK 293 cells was close to the background level (~8%; Table 1).

In sum, cytotoxicity assays have confirmed the high tissue-specificity of mNUS-RCD cassette, because cytotoxicity was strong in Tera-1 cells, but undetectable in HEK293 cells. Accordingly, it is likely that no cytotoxic effect may be seen in all other above cell cultures, as the promoter activity of mNUS was lower in them than in HEK293.

### Tissue-specific transposition of *Sleeping Beauty *by mNUS-specific transposase expression

To assess the applicability of the mNUS promoter for tissue-specific transgene integration, we employed a transposon-based system. DNA transposons are mobile elements capable to move in and into genomes using a self-encoded protein termed transposase. The transposase recognizes so-called inverted terminal repeat (ITR) sequences that flank each copy of DNA transposon, and mediates physical excision of a transposon copy with further re-insertion into a new location whithin the genome [21,22]. Nowadays, transposons are widely used by researchers for stable gene delivery [23], in which transgene cassettes flanked by the transposon-specific ITRs, are mobilized and stably integrate into the genomes of cells, in which the transposase is expressed (*e.g*., transcribed from a cotransfected helper vector).

We used a stable gene delivery system based on the *Sleeping Beauty *(SB) DNA transposon to further validate the tissue-specific properties of the mNUS promoter. High efficiency of its transposase, recently enhanced more than 100-fold by directed mutagenesis [24], and random chromosomal insertion make the SB system a method of choice for many applications [25].

**Figure 3:**
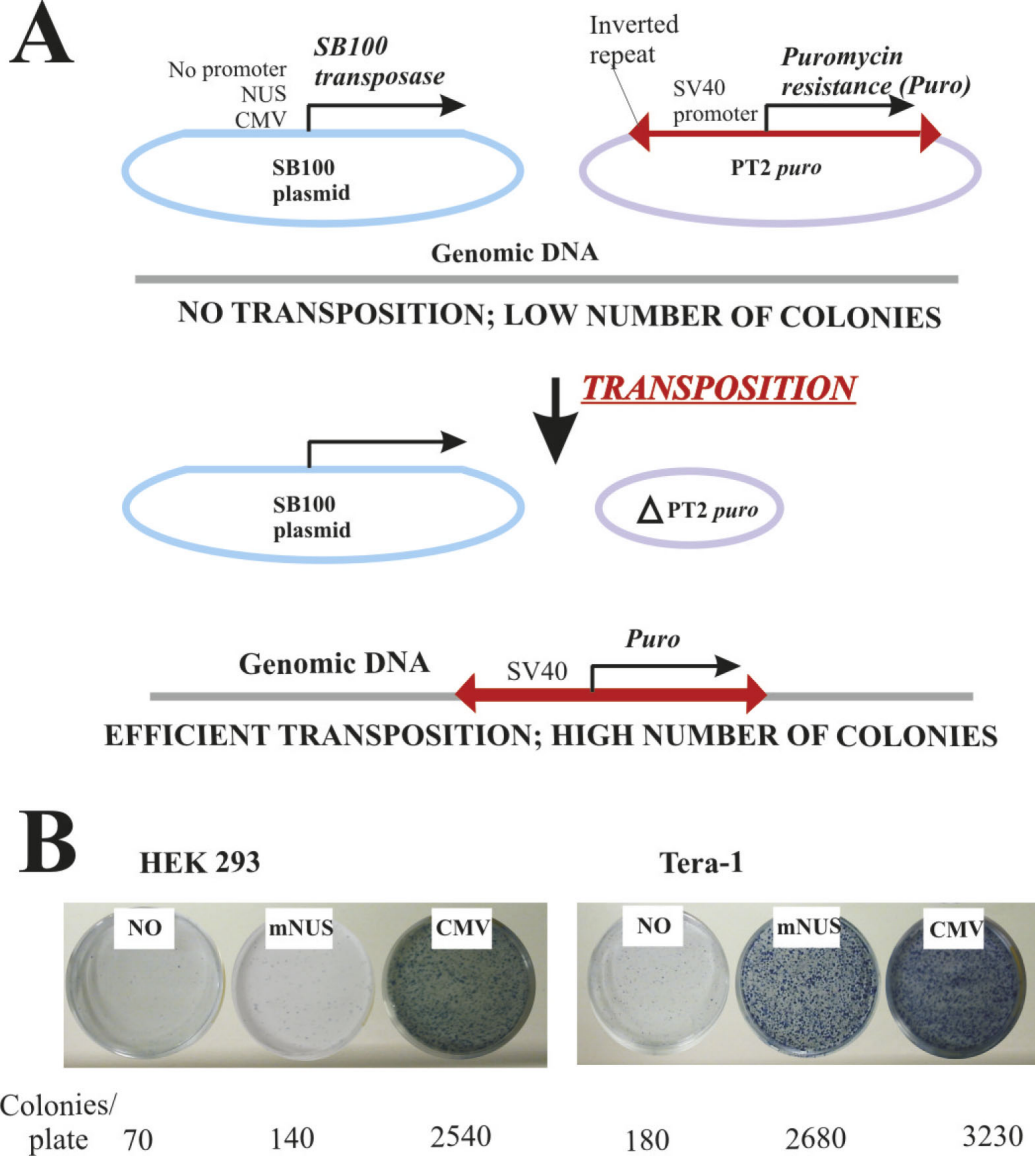
**Comparison of mNUS activity in Tera-1 and HEK293 cell lines in *Sleeping Beauty *transposition**. (A) Overall scheme of the transposition assay. Only those cells that receive a genomic insertion of a puromycin resistance cassette may grow and form colonies when exposed to antibiotic. In case of random, non-SB100X-mediated genomic integrations, the number of colonies is relatively low, whereas when SB100X transposase gene is active the number of colonies is significantly higher. (B) Representative colony number screens. Average colony numbers for each type of experiment are shown below the representative plates.

The enhanced SB transposase (SB100X) was transiently expressed in transfected cells under the control of mNUS promoter. The positive control vector had CMV promoter-driven SB100X, whereas a promoterless SB100X cassette served as negative control. SB100X vectors were cotransfected with a plasmid harboring a puromycin resistance (puro) cassette flanked by the SB ITRs (Fig. 3). Expression of SB100X mediates high number of transposition events that result in a puromycin-resistant phenotype of the cells. In our assay, we cotransfected Tera-1 and HEK293 cells with the pairs of *SB100X*-harboring vector plus ITR-*Puro *plasmid. In HEK293 cells, the numbers of colonies seen for mNUS-SB100X was very similar to those obtained in the negative control transfectants, suggesting lack of transposition. High colony numbers in the positive control indicate efficient transposition (Fig. 3B). In Tera-1 cells, the number of colonies was low in the negative control, high in the positive control, and also high in case of mNUS-SB100X transfectants (~80% of the positive control colony number).

These results suggest that mNUS-based constructs may be efficiently used for the tissue-specific stable gene delivery. This is also in line with our previous findings that in Tera-1 cells mNUS promoter strength is comparable to that of the CMV promoter.

## Discussion

In this paper we report a novel tissue-specific promoter, mNUS, that is highly active in human undifferentiated testicular germ cancer cells (lines Tera-1 and EP2102), but has no apparent activity in the other tested cell types. The potential applicability of this promoter element was illustrated in two examples.

First, we designed a suicide gene therapy cassette including mNUS as an upstream transcriptional regulatory element, and the gene for recombinant cytosine deaminase (RCD) as a cytotoxic agent. This mNUS-RCD cassette has demonstrated strong, tissue-specific cytotoxic effect restricted to germ cancer cells. We hope that high tissue specificity and low background toxicity of the mNUS-based cassette may be useful for the design of novel efficient therapeuticals against germ cell tumors (*e.g*., seminomas).

Second, the mNUS promoter was applied for generating a tissue-specific system for stable gene delivery. The *Sleeping Beauty *transposase expressed under transcriptional control of mNUS efficiently mediated stable gene transfer into genomes of germ cells, but not in somatic control cells.

The mNUS promoter element is rather large (~3.6 kb), which may limit its usefulness. However, as we show here it may be efficiently applied for both reporter constructs, gene transfer and suicide gene therapy vectors. We hope that apart from these applications, mNUS may be utilized as a tool for studying development, proliferation and functional analyses of germ cells and related tissues.

Our data also indicate that the major upstream regulatory elements of the native *NDUFV1 *promoter are likely to be concentrated within the 91 bp-long region located just upstream the transcriptional start site. Here we present a map of putative transcription factor binding sites (TFBS) identified within NUS, predicted using the TRANSFAC database (Additional file 4, Fig. S4). Indeed, the above 91 bp-long region is enriched in putative TFBS, as compared to the upstream sequence. The second region where the highest concentration of TFBS occurs is located within the endogenous retroviral HERV-K(HML-2) LTR sequence. Therefore, the endogenous retroviral sequence likely plays an important role in mNUS promoter activity. This hypothesis is consistent with our further results showing that *in vitro *promoter activity of mNUS in Tera-1 and EP2102 cell lines was ~3- and ~4-times decreased when the HERV-K(HML-2) LTR sequence was removed from mNUS (unpublished data). Finally, tissue-specificity of the mNUS promoter may be due to the presence of SRY/SOX3 transcriptional factor recognition motifs that account, among others, for germ cell-specific transcriptional upregulation of gammaretroviruses and LINE-1 retrotransposons. Seven such motifs have been identified within the mNUS HERV-K(HML-2) LTR, and six within the downstream mNUS sequence (Fig. S4). Alternatively, LTR enhancer activity at least in part may be driven by nuclear factor OCT3/4. This protein is known as a marker of pluripotency, that plays a pivotal role in the early stages of normal development in mammals. It was found also to be the most informative diagnostic marker for the early stages of malignant germ cell tumors. The LTR contains three OCT3/4 binding motifs, and the downstream mNUS regulatory sequence - two additional ones. Experimental functional analysis of these sites and further attempts to shorten the mNUS sequence will be subject of future studies.

## Conclusions

We conclude that mNUS may be used as an efficient promoter for tissue specific gene expression in human germ cells in various applications. Our data also suggest that the 91 bp-long sequence located exactly upstream *NDUFV1 *transcriptional start site plays a crucial role in the activity of this gene promoter *in vitro *in the majority of tested cell types (10/12), and an important role - in the rest two cell lines.

## Methods

### Cloning of *NDUFV1 *upstream regions

*NDUFV1 *upstream region was explored using UCSC Human Genome Browser http://genome.ucsc.edu/. Native *NDUFV1 *upstream sequence (NUS) and modified *NDUFV1 *upstream sequence (mNUS) were PCR-amplified using 40 ng of human genomic DNA. For NUS amplification, the following oligonucleotides were used (5'-3'): NDfor, *ACGCGTTGAGTATGCTGCAGGCTTGG*; NDrev1, *AGATCTCACTGAGGCTGAGGAACTGG*. mNUS was amplified using the above NDfor primer in pair with the primer NDrev2 (*AGATCTGCTGCGCCCCTTCAACTTCGCC*). PCR conditions for both amplifications were as follows: 95°C - 2 min; 95°C - 25 s, 62°C - 25 s, 72°C - 4 min; 30 cycles. PCR products (lengths 3665 and 3574 bp for NUS and mNUS, respectively) were cloned into pGL3 basic vector (Promega) upstream the reporter gene for Luciferase using *MluI *and *BglII *restriction sites. Primary structure of the cloned inserts and their 100% identity to the reference genomic sequence (human genomic contig NT_167190.1, positions 12676563-12680227 and 12676563-12680136, respectively) were confirmed by sequencing.

### Cell cultures

The following established human cell lines were used: EP2102, Tera1 (testicular embryonal germ cell tumors [26]), Tera-2 (partly differentiated testicular germ cells with characteristics of central nervous system precursor cells), NGP127 (neuroblastoma cells), HepG2 (hepatocarcinoma), A549 (lung carcinoma) and HEK 293 (transformed embryonic kidney cells). All cell lines except EP2102 were kindly provided by Dr. Sergey Akopov and Dr. Eugene Kopanzev (Shemyakin-Ovchinnikov Institute of Bioorganic Chemistry). EP2102 cells were obtained from the collection of Max Delbrück Center for Molecular Medicine. Primary human cell cultures were as follows: human peripheral blood macrophages, provided by Dr. Olga Zatsepina (Shemyakin-Ovchinnikov Institute of Bioorganic Chemistry); umbilical cord mesenchymal stem cells (UC-MSC), placental mesenchymal stem cells (PMSC) and bone marrow mesenchymal stem cells (BM-MSC), fetal brain cells (FBC) provided by Dr. Viktor Sorokin and Prof. Konstantin Yarygin (Russian State Medical University). All subjects gave consent for use of the above mentioned human cells in this research. The cells were grown in DMEM/F12 (1:1) medium containing 10% fetal calf serum (Invitrogen) at 37°C and 5% CO_2_.

### Luciferase activity assay

Transfections were carried out in 24-well plates using Lipofectamine 2000 (Invitrogen) according to the manufacturer recommendations. Dual luciferase system (Promega) was used for the luciferase activity screens. For each transfection, 0.5 mcg of 10:1 mixture of the analytical plasmid (including firefly luciferase under control of the regulatory sequence of interest) and normalization plasmid was used. We used pRL-TK normalization vector (Promega) including another reporter gene - *Renilla reniformis *luciferase, under control of a herpes simplex virus thymidine kinase promoter to provide relatively uniform levels of *Renilla *luciferase expression in co-transfected cells. Prior transfections with analytical plasmids, we have tested thymidine kinase promoter -driven *Renilla *luciferase expression in normalization vector on five human cell cultures. *Renilla *luciferase activity was measured in the cell cultures Tera-1, NGP127, HepG2, A549 and HEK293 transfected with pRL-TK normalization vector. The luciferase activity was scored only for the experiments with transfection efficiencies 65-70%. Transfection efficiencies were measured by the GFP fluorescence when cotransfecting pRL-TK (Promega) with pTurbo-GFP plasmid (Evrogen). Our results confirm the data from Promega dual luciferase manual that pRL-TK may serve as an efficient normalization vector (Fig. S2).

Analytical plasmids based on pGL3 vector (Promega) had the following regulatory sequences: cloned NUS or mNUS, or SV40 early promoter. 24-Hours after transfection, cells were lysed and the activities of *Renilla *and firefly luciferases were measured using Dual-Luciferase Reporter Assay System (Promega) using luminometer «GENios Pro» (Tecan). Plasmid pRL-TK was used in all experiments as the internal control to minimize errors caused by the differences in transfection efficiencies in independant replicates. The obtained values for the firefly luciferase were normalized to the values for the *Renilla *luciferase. Each transfection experiment was done at least in quadruplicate.

### Cloning of the gene for recombinant cytosine deaminase

Bifunctional recombinant cytosine deaminase (RCD) termed "*SuperCD *suicide fusion gene" in previous report [20] was generated according to the published protocol. Bifunctional *RCD *gene was cloned into the plasmids pCI and pGL3 basic (Promega) linearized by *Not*I-*Sal*I. This resulted in plasmids carrying gene *RCD *either under the control of standard cytomegaloviral (CMV) promoter (pCI-CMV-RCD) or without any upstream regulatory elements (pGL3-no-RCD). mNUS fragment was cloned into pGL3-no-RCD using *Hind*III and *Not*I restriction sites. *RCD *identity in all constructs was confirmed by complete sequencing.

### Western blot analysis

Cells were lysed in SDS-containing sample buffer. Lysates were separated in 10% SDS-PAGE and transferred to PVDF membranes. Polyclonal sheep antibody against a fragment of RCD molecule (Abcam, USA) was used to detect RCD expression. For control experiments, monoclonal anti-GAPDH antibodies (Sigma) were used. Immunoreactive bands were visualized using anti-sheep secondary goat IgG-horseradish peroxidase conjugate (Promega, USA) and ECL detection reagents (Biorad, Russia).

### Cell viability tests

Cells were transfected in 6-well plates with 10:1 mixture of the RCD-containing vector and pTurbo-GFP (Evrogen) using Lipofectamine 2000 (Invitrogen) according to the manufacturer recommendations. pTurbo-GFP served as normalization plasmid carrying GFP gene to estimate transfection efficiencies (only transfections with the efficiency values 65-80% were scored). Approximately 12 hours after transfections, 500 μM 5FC (5-fluoro cytosine) containing medium was added to the cells (this 5FC concentration was reported to be the optimum for RCD assays [19,20]). 5-FC was purchased from Sigma (St. Louis, MO). Viable cells were counted 24 hours and 48 hours after addition of the 5FC. Each experiment was done in quadruplicate for the PI assay and in three replicas - for the Annexin V test. Propidium iodide assay with subsequent FACS data analysis were performed according to the standard protocol (*e.g*., as described in [27,28]). Annexin V assay and flow cytometric quantization of dead cells were carried out using the Annexin V kit (Caltag Laboratories) according to the manufacturers' protocol using Coulter EPICS XL flow cytometer (Beckman Coulter).

### Transposition assay

Cells were split ~3 days before transfection and reached between 70-100% confluency. Transfections were done using Unifectin 2000 and FuGENE (Roche) transfection reagents according to the manufacturer recommendations. For transfections, the following plasmid vectors were used: pT2B/*puro *including puromycin resistance gene under control of SV40 promoter flanked by the *Sleeping Beauty *inverted repeats; CMV-SB100X vector harboring enhanced *Sleeping Beauty *transposase gene under control of CMV promoter (courtesy of Dr. Lajos Mates, Max-Delbruck Center for Molecular Medicine, Berlin, Germany); no-SB100X vector without promoter sequence, mNUS-SB100X vector with mNUS promoter placed upstream of the SB100X gene, and TurboGFP vector (Evrogen) including GFP gene. Transfection efficiency was assessed by GFP fluorescence. The following vector combinations were used for transposition assays: a) pT2B/*puro *alone, b) pT2B/*puro *+ no-SB100X vector, c) pT2B/*puro *+ CMV-SB100X, d) pT2B/*puro *+ mNUS-SB100X. After transfections, cells were incubated at 37C, 5% CO2 for 48 hours. The medium was then removed and the cells were washed with PBS, followed by 1-minute trypsinization, stopped by the addition of 500 ul of serum-containing cell culture media to each well. Different volumes of cells mixture (50%, 20% and 10%) were plated out on 10 cm Petri dishes for further puromycin selection (~7 days). 10 ml of puromycin -containing medium were added to each dish. We changed medium every two days until selection was complete and distinct colonies formed (2-3 mm diameter). To stain the colonies, the medium was aspirated and plates were washed with PBS. Each plate was fixed in 10% formaldehyde in PBS and was incubated for 20 min at room temperature under chemical fume. The fixing solution was then removed and plates were washed twice with 10 ml of PBS. 5 ml of staining solution (0,5% methylene blue in PBS) was added and plates were incubated for 30 min or more. The dye was further removed and plates were washed in bucket of cold water. The plates were allowed to dry, be photographed and analyzed. Transposition efficiency was determined as the ratio of colonies formed in the presence of transposase versus colony numbers in the absence of transposase formed due to random integration of antibiotic resistance cassette.

## Authors' contributions

DK carried out experiments on RCD cloning, did several NUS/mNUS constructs and performed cell viability tests; EG cloned NUS and mNUS variants, did luciferase activity screens; RK did cell viability tests; DPG did transposition assay and wrote the paper; MM did luciferase activity screens; TV and EK took part in RCD cloning and functional tests; GM did cell culture work and performed luciferase activity screens; MS did bioinformatical analysis; DS obtained primary cell cultures; ZI participated in design of the study and wrote the manuscript; AB did several mNUS constructs, carried out transposition assay, coordinated efforts of the joint research team and wrote the paper. All authors read and approved the final manuscript.

## Supplementary Material

Additional file 1 **Figure S1: Schematic representation of *NDUFV1 *upstream region.** 3665 bp-long NUS sequence contains highly conserved 91 bp-long genomic fragment located exactly upstream *NDUFV1 *transcriptional start site. At the 5' terminus of NUS there is an insert of HERV-K (HML-2) solitary LTR. Click here for file

Additional file 2 **Figure S2: Comparison of herpes virus thymidine kinase promoter - driven *renilla *luciferase activities in cell lines Tera-1, NGP127, HepG2, A549 and HEK293.** The column height reflects the mean value of detected luciferase activity from at least six transfection experiments, and error bars indicate the value of a standard error. Each experiment was made at least in quadruplicate. Click here for file

Additional file 3 **Figure S3: Comparison of mNUS and CMV promoter activities in cell lines Tera-1 and HEK293. **The column height reflects the mean value of relative luciferase activity from at least four transfections, and error bars indicate the value of a standard error. Click here for file

Additional file 4 **Figure S4: Schematic map of the putative transcriptional factor binding sites within the 3665 bp-long entire NUS region.** HERV-K(HML-2) LTR sequence and 91 bp-long deletion are shown by magenta boxes. Signs above/below represent putative transcription factor binding sites identified in the sense/antisense orientations, respectively. Large red circle represent putative SRY/SOX3 binding site perfectly matching the consensus sequence (ACAAAACA), small circles - sites with single nucleotide mismatches. The detailed data on transcriptional factor binding sites is presented on the additional table 1. Click here for file

## References

[B1] Dong Z Nor JE Transcriptional targeting of tumor endothelial cells for gene therapy Adv Drug Deliv Rev 2009 61 7-8 542 553 10.1016/j.addr.2009.02.006 19393703 PMC2727054

[B2] Hebrard C Dumontet C Jordheim LP Development of gene therapy in association with clinically used cytotoxic deoxynucleoside analogues Cancer Gene Ther 2009 16 7 541 550 10.1038/cgt.2009.25 19343063

[B3] Lian JB Stein GS Stein JL van Wijnen AJ Marrow transplantation and targeted gene therapy to the skeleton Clin Orthop Relat Res 2000 379 Suppl S146 155 10.1097/00003086-200010001-00019 11039763

[B4] Vilalta M Degano IR Bago J Aguilar E Gambhir SS Rubio N Blanco J Human adipose tissue-derived mesenchymal stromal cells as vehicles for tumor bystander effect: a model based on bioluminescence imaging Gene Ther 2009 16 4 547 557 10.1038/gt.2008.176 19092860

[B5] Vigna E Pacchiana G Mazzone M Chiriaco C Fontani L Basilico C Pennacchietti S Comoglio PM "Active" cancer immunotherapy by anti-Met antibody gene transfer Cancer Res 2008 68 22 9176 9183 10.1158/0008-5472.CAN-08-1688 19010889

[B6] Song J Kim C Ochoa ER Sleeping Beauty-mediated suicide gene therapy of hepatocellular carcinoma Biosci Biotechnol Biochem 2009 73 1 165 168 10.1271/bbb.80581 19129627

[B7] Kucerova L Matuskova M Pastorakova A Tyciakova S Jakubikova J Bohovic R Altanerova V Altaner C Cytosine deaminase expressing human mesenchymal stem cells mediated tumour regression in melanoma bearing mice J Gene Med 2008 10 10 1071 1082 10.1002/jgm.1239 18671316

[B8] Baust C Seifarth W Schon U Hehlmann R Leib-Mosch C Functional activity of HERV-K-T47D-related long terminal repeats Virology 2001 283 2 262 272 10.1006/viro.2001.0898 11336551

[B9] Schon U Diem O Leitner L Gunzburg WH Mager DL Salmons B Leib-Mosch C Human endogenous retroviral long terminal repeat sequences as cell type-specific promoters in retroviral vectors J Virol 2009 83 23 12643 12650 10.1128/JVI.00858-09 19741000 PMC2786743

[B10] Bannert N Kurth R Retroelements and the human genome: new perspectives on an old relation Proc Natl Acad Sci USA 2004 101 Suppl 2 14572 14579 10.1073/pnas.0404838101 15310846 PMC521986

[B11] Buzdin A Kovalskaya-Alexandrova E Gogvadze E Sverdlov E At least 50% of human-specific HERV-K (HML-2) long terminal repeats serve in vivo as active promoters for host nonrepetitive DNA transcription J Virol 2006 80 21 10752 10762 10.1128/JVI.00871-06 17041225 PMC1641792

[B12] Buzdin A Human-specific endogenous retroviruses ScientificWorldJournal 2007 7 1848 1868 18060323 10.1100/tsw.2007.270 PMC5901341

[B13] Lavie L Kitova M Maldener E Meese E Mayer J CpG methylation directly regulates transcriptional activity of the human endogenous retrovirus family HERV-K(HML-2) J Virol 2005 79 2 876 883 10.1128/JVI.79.2.876-883.2005 15613316 PMC538560

[B14] Ruda VM Akopov SB Trubetskoy DO Manuylov NL Vetchinova AS Zavalova LL Nikolaev LG Sverdlov ED Tissue specificity of enhancer and promoter activities of a HERV-K(HML-2) LTR Virus Res 2004 104 1 11 16 10.1016/j.virusres.2004.02.036 15177887

[B15] Ruprecht K Ferreira H Flockerzi A Wahl S Sauter M Mayer J Mueller-Lantzsch N Human endogenous retrovirus family HERV-K(HML-2) RNA transcripts are selectively packaged into retroviral particles produced by the human germ cell tumor line Tera-1 and originate mainly from a provirus on chromosome 22q11.21 J Virol 2008 82 20 10008 10016 10.1128/JVI.01016-08 18684837 PMC2566279

[B16] Sverdlov ED Retroviruses and primate evolution Bioessays 2000 22 2 161 171 10.1002/(SICI)1521-1878(200002)22:2<161::AID-BIES7>3.0.CO;2-X 10655035

[B17] Ali ST Duncan AM Schappert K Heng HH Tsui LC Chow W Robinson BH Chromosomal localization of the human gene encoding the 51-kDa subunit of mitochondrial complex I (NDUFV1) to 11q13 Genomics 1993 18 2 435 439 10.1006/geno.1993.1493 8288251

[B18] Andrews PW Teratocarcinomas and human embryology: pluripotent human EC cell lines. Review article Apmis 1998 106 1 158 167 discussion 167-158 10.1111/j.1699-0463.1998.tb01331.x 9524574

[B19] Erbs P Regulier E Kintz J Leroy P Poitevin Y Exinger F Jund R Mehtali M In vivo cancer gene therapy by adenovirus-mediated transfer of a bifunctional yeast cytosine deaminase/uracil phosphoribosyltransferase fusion gene Cancer Res 2000 60 14 3813 3822 10919655

[B20] Graepler F Lemken ML Wybranietz WA Schmidt U Smirnow I Gross CD Spiegel M Schenk A Graf H Lauer UA Vonthein R Gregor M Armeanu S Bitzer M Lauer UM Bifunctional chimeric SuperCD suicide gene -YCD: YUPRT fusion is highly effective in a rat hepatoma model World J Gastroenterol 2005 11 44 6910 6919 16437592 10.3748/wjg.v11.i44.6910 PMC4717030

[B21] Buzdin A Transposable elements and their use for target site specific gene delivery Current Pharmacogenomics 2006 4 1 1 8 10.2174/157016006776055437

[B22] Ryder E Russell S Transposable elements as tools for genomics and genetics in Drosophila Brief Funct Genomic Proteomic 2003 2 1 57 71 10.1093/bfgp/2.1.57 15239944

[B23] Izsvak Z Ivics Z Sleeping beauty transposition: biology and applications for molecular therapy Mol Ther 2004 9 2 147 156 10.1016/j.ymthe.2003.11.009 14759798

[B24] Mates L Chuah MK Belay E Jerchow B Manoj N Acosta-Sanchez A Grzela DP Schmitt A Becker K Matrai J Ma L Samara-Kuko E Gysemans C Pryputniewicz D Miskey C Fletcher B Vandendriesshe T Ivics Z Izsvak Z Molecular evolution of a novel hyperactive Sleeping Beauty transposase enables robust stable gene transfer in vertebrates Nat Genet 2009 41 6 753 761 10.1038/ng.343 19412179

[B25] Ivics Z Li MA Mates L Boeke JD Nagy A Bradley A Izsvak Z Transposon-mediated genome manipulation in vertebrates Nat Methods 2009 6 6 415 422 10.1038/nmeth.1332 19478801 PMC2867038

[B26] Jewett MA Testis carcinoma: transplantation into nude mice Natl Cancer Inst Monogr 1978 49 65 66 748798

[B27] Kholodenko R Kholodenko I Sorokin V Tolmazova A Sazonova O Buzdin A Anti-apoptotic effect of retinoic acid on retinal progenitor cells mediated by a protein kinase A-dependent mechanism Cell Res 2007 17 2 151 162 10.1038/sj.cr.7310147 17297481

[B28] Telford WG Komoriya A Packard BZ Multiparametric analysis of apoptosis by flow and image cytometry Methods Mol Biol 2004 263 141 160 14976365 10.1385/1-59259-773-4:141

