# Supplementary figures and images for: Novel strong tissue specific promoter for gene expression in human germ cells
Source: BMC Biotechnol. 2010 Aug 17;10:58. doi: 10.1186/1472-6750-10-58 (PMC2929213; doi:10.1186/1472-6750-10-58)

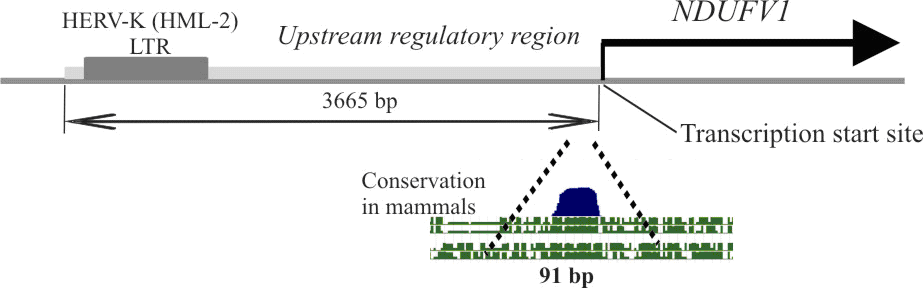

Supplement: Additional file 1 — Figure S1: Schematic representation of NDUFV1 upstream region. 3665 bp-long NUS sequence contains highly conserved 91 bp-long genomic fragment located exactly upstream NDUFV1 transcriptional start site. At the 5' terminus of NUS there is an insert of HERV-K (HML-2) solitary LTR. [file 1472-6750-10-58-S1.TIFF]

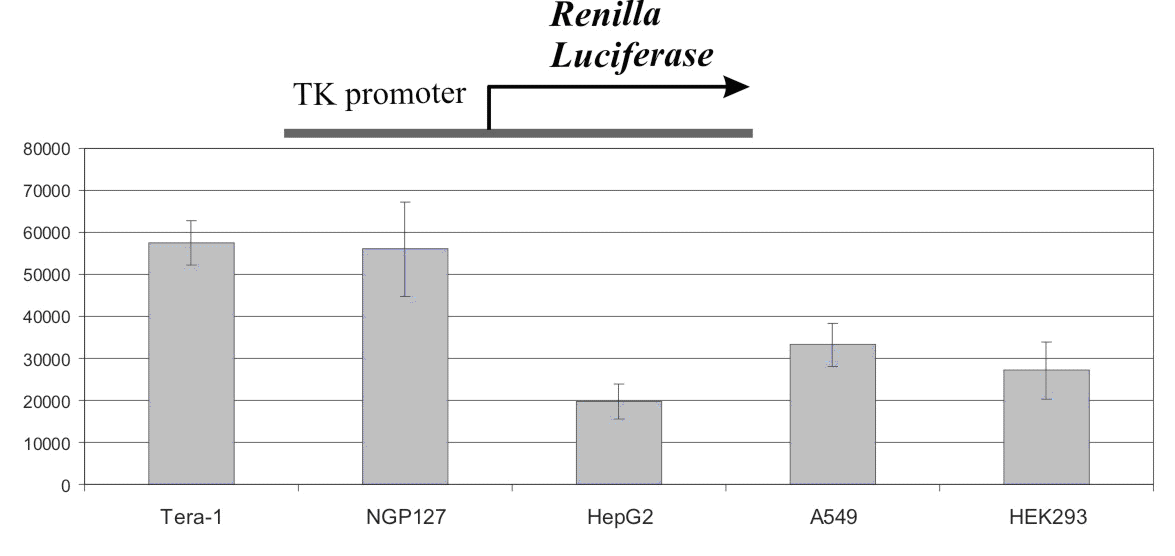

Supplement: Additional file 2 — Figure S2: Comparison of herpes virus thymidine kinase promoter - driven renilla luciferase activities in cell lines Tera-1, NGP127, HepG2, A549 and HEK293. The column height reflects the mean value of detected luciferase activity from at least six transfection experiments, and error bars indicate the value of a standard error. Each experiment was made at least in quadruplicate. [file 1472-6750-10-58-S2.TIFF]

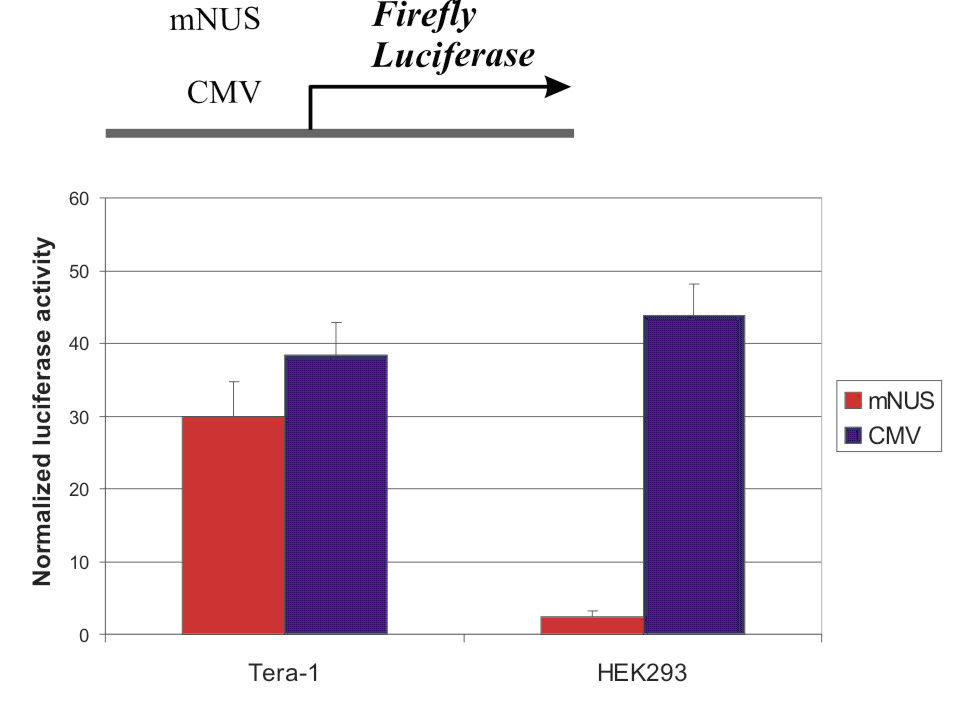

Supplement: Additional file 3 — Figure S3: Comparison of mNUS and CMV promoter activities in cell lines Tera-1 and HEK293. The column height reflects the mean value of relative luciferase activity from at least four transfections, and error bars indicate the value of a standard error. [file 1472-6750-10-58-S3.TIFF]

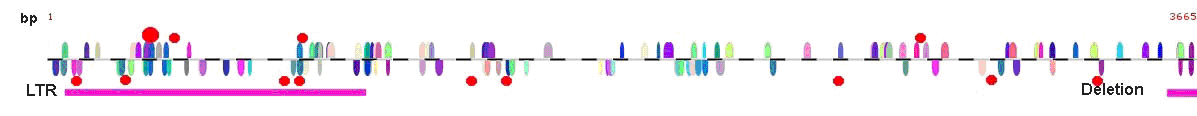

Supplement: Additional file 4 — Figure S4: Schematic map of the putative transcriptional factor binding sites within the 3665 bp-long entire NUS region. HERV-K(HML-2) LTR sequence and 91 bp-long deletion are shown by magenta boxes. Signs above/below represent putative transcription factor binding sites identified in the sense/antisense orientations, respectively. Large red circle represent putative SRY/SOX3 binding site perfectly matching the consensus sequence (ACAAAACA), small circles - sites with single nucleotide mismatches. The detailed data on transcriptional factor binding sites is presented on the additional table 1. [file 1472-6750-10-58-S4.TIFF]
